# Functional Divergence Caused by Ancient Positive Selection of a *Drosophila* Hybrid Incompatibility Locus

**DOI:** 10.1371/journal.pbio.0020142

**Published:** 2004-06-15

**Authors:** Daniel A Barbash, Philip Awadalla, Aaron M Tarone

**Affiliations:** **1**Section of Evolution and Ecology, University of CaliforniaDavis, CaliforniaUnited States of America; **2**Department of Genetics, North Carolina State UniversityRaleigh, North CarolinaUnited States of America

## Abstract

Interspecific hybrid lethality and sterility are a consequence of divergent evolution between species and serve to maintain the discrete identities of species. The evolution of hybrid incompatibilities has been described in widely accepted models by Dobzhansky and Muller where lineage-specific functional divergence is the essential characteristic of hybrid incompatibility genes. Experimentally tractable models are required to identify and test candidate hybrid incompatibility genes. Several Drosophila melanogaster genes involved in hybrid incompatibility have been identified but none has yet been shown to have functionally diverged in accordance with the Dobzhansky-Muller model. By introducing transgenic copies of the X-linked *Hybrid male rescue (Hmr)* gene into D. melanogaster from its sibling species D. simulans and *D. mauritiana,* we demonstrate that *Hmr* has functionally diverged to cause F1 hybrid incompatibility between these species. Consistent with the Dobzhansky-Muller model, we find that *Hmr* has diverged extensively in the D. melanogaster lineage, but we also find extensive divergence in the sibling-species lineage. Together, these findings implicate over 13% of the amino acids encoded by *Hmr* as candidates for causing hybrid incompatibility. The exceptional level of divergence at *Hmr* cannot be explained by neutral processes because we use phylogenetic methods and population genetic analyses to show that the elevated amino-acid divergence in both lineages is due to positive selection in the distant past—at least one million generations ago. Our findings suggest that multiple substitutions driven by natural selection may be a general phenomenon required to generate hybrid incompatibility alleles.

## Abstract 2

Transgenic experiments show that the HMR gene has functionally diverged in Drosophila melanogaster and its sibling species and causes the death of hybrid offspring in interspecific crosses

## Introduction

**Figure 1:**
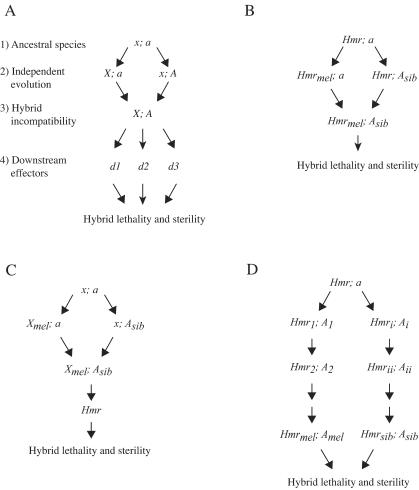
Models of Hybrid Incompatibility (A) D-M model of HI evolution. We diagram here an X–autosome incompatibility; for simplicity only haploid genotypes are shown. This model can be easily extended to include more complex multilocus interactions. (1) The ancestral species is fixed for the X-linked allele *x* and the autosomal allele *a*. (2) As the two species independently diverge, one becomes fixed for allele *X* at the first locus and the other for allele *A* at the second locus. (3) HI is caused by the interaction between these derived alleles, *X* and *A.* (4) This interaction may cause misregulation of downstream effector genes *(d1, d2,* and *d3),* which in turn causes the HI phenotype. (B) *Hmr* is an HI gene. *Hmr_mel_* has evolved in the D. melanogaster lineage and interacts to cause HI with *A_sib_,* an allele of a hypothesized autosomal gene that has evolved in the sibling-species lineage. Mutations in *Hmr_mel_* allow hybrid viability by eliminating the activity of this incompatibility allele. (C) *Hmr* is a downstream effector gene. Here, two unknown genes cause HI by misregulating *Hmr.* Mutations in *Hmr* allow hybrid viability by acting as downstream suppressors of the HI alleles. (D) Model with *Hmr* and gene *A* extensively diverging (see Discussion). Both *Hmr* and gene *A* coevolve with many changes along both lineages. HI could be caused by interactions between derived alleles or between a derived and an ancestral allele. All models to identify the codons in *Hmr* responsible for functional divergence have two constraints: first, that *Hmr* and gene *A* must be fully compatible with each other in each lineage, and second, that candidate codons must differ between *Hmr_sib_* from all three sibling species and *Hmr_mel_.*

Reproductive isolation is the most commonly used criterion to define species. Hybrid incompatibilities (HIs) such as hybrid sterility and lethality are widely observed examples of reproductive isolation. The Dobzhansky-Muller (D-M) model explains how the genes causing deleterious phenotypes in hybrids can evolve (Dobzhansky 1937; Muller 1942; Turelli and Orr 2000) (Figure 1A). The model holds that HIs arise from the interaction between two or more genes that have evolved independently in two isolated populations; the deleterious phenotypes caused by these genes are a by-product of intraspecific divergence and will occur only when the genes interact in the interspecific hybrid. The essential criterion for defining HI genes, therefore, is that the alleles from the two species have distinct phenotypic properties in hybrids: for example, in Figure 1A the derived allele *A* from one species causes the incompatibility while the ancestral allele *a* from the other species does not.

This model makes clear and testable genetic predictions, namely, that experimental manipulation of allele *A* (or *X*) but not allele *a* (or *x*) will affect the HI phenotype. For example, increasing the dosage or activity of allele *A* should decrease hybrid fitness, while identical manipulations of allele *a* should not. In contrast, downstream effector genes such as *d1* in Figure 1A contribute to the phenotype of HI but are not expected to have functionally diverged alleles in the two hybridizing species; in other words, experimental manipulation of downstream effector alleles from either species will have equivalent effects on the HI phenotype. These alternative possibilities can only be addressed by genetically manipulating each species allele in a controlled hybrid background, but this has yet to be achieved in model organisms such as *Drosophila melanogaster.*

If the genetic changes that cause HI are rare, then genes that have undergone extensive divergence may be more likely to cause HI simply because there is more chance that they have experienced rare HI-causing mutations. But the D-M model itself offers no suggestions about the mode of evolution that leads to this divergence. One possibility is that HI genes accumulate genetic changes over time as they evolve neutrally. Alternatively, many models have suggested that speciation may be driven by molecules that are undergoing natural selection, for example, under the pressure of ecological divergence (Schluter 2001) or sexual selection (Lande 1981; Parker and Partridge 1998). If genes associated with HI evolve under positive selection, one consequence is that they may be disproportionately X-linked, as positively selected, recessive X-chromosome alleles may go to fixation more quickly and therefore cause X-linked loci to evolve more rapidly relative to autosomal loci (Charlesworth et al. 1987). This preferential X-linkage may contribute to Haldane's rule (Haldane 1922), the observation that heterogametic (e.g., XY) hybrids suffer more incompatibilities than homogametic (XX) hybrids. A second consequence of selection is that HI genes may have unique phylogenetic and population genetic signatures (Wang et al. 1997; Ting et al. 2000). Molecular evolutionary analyses of speciation genes are essential to address the role of selection in reproductive isolation and speciation.

D. melanogaster can hybridize with three closely related species that we refer to collectively as its sibling species: *D. simulans, D. mauritiana,* and *D. sechellia.* Crosses between D. melanogaster females and sibling-species males result in invariantly lethal hybrid sons and temperature-dependent lethal hybrid daughters (Sturtevant 1920; Lachaise et al. 1986; Barbash et al. 2000). The *Hybrid male rescue (Hmr)* gene has a major effect on the fitness of hybrids from this cross. This point is most strikingly demonstrated by the fact that *D. melanogaster Hmr* loss-of-function mutations such as *Hmr^1^* suppress the lethality of both hybrid males and females (Hutter and Ashburner 1987; Barbash et al. 2000), as does the X-linked *In(1)AB* rescue mutation (Hutter et al. 1990). Increasing the dosage of the wild-type gene *Hmr^+^* has the reciprocal property of decreasing hybrid viability (Barbash et al. 2000; Orr and Irving 2000). These studies led to the proposal that HI is caused by an interaction between the X-linked *D. melanogaster Hmr^+^* gene and an unknown autosomal gene(s) from the sibling species. Barbash and colleagues (2003) recently cloned the *Hmr* gene, which encodes a predicted DNA-binding protein similar to the ADF and MYB family of transcriptional regulators, and proposed that D. melanogaster HI may be caused by transcriptional misregulation.

A limitation of previous genetic analyses of *Hmr* is that all genetic manipulations were done only with the D. melanogaster allele. These studies therefore cannot determine whether *Hmr* is an HI gene as modeled by Dobzhansky and Muller (Figure 1B) or is instead a downstream effector gene that suppresses hybrid lethality because it interacts with or is regulated by the actual HI genes (Figure 1C). Similar uncertainties apply to the *D. melanogaster Zhr* and *Nup96* genes (Sawamura et al. 1993; Presgraves et al. 2003), which also affect F1 hybrid viability.

A preliminary analysis suggested that *Hmr* is highly diverged between D. melanogaster and the sibling species, with almost 8% divergence at nonsynonymous (amino-acid replacement) sites, a remarkable finding considering that *Hmr* encodes a predicted protein over 1,400 amino acids in length. Two other *Drosophila* genes that are involved in HI show elevated divergence that appears to have evolved under positive selection, but the divergence is confined to a small region of each gene (Ting et al. 1998; Presgraves et al. 2003) and *Hmr* is two times more diverged than either locus. These differences raise the question of whether the extensive divergence of *Hmr* may instead have evolved neutrally, or whether there are regional differences in selection and divergence at *Hmr.*

In this study we use transgenic assays to test whether *Hmr* has functionally diverged between D. melanogaster and its sibling species and thus fits the D-M model of HI evolution. We examine the functional consequences of this divergence and discuss models to determine which lineages and which codons have undergone functional divergence. We also test whether *Hmr* has evolved by positive selection and determine the number and locations of regions and codons that show particularly strong signals of adaptive evolution, asking whether the patterns of polymorphism at *Hmr* are consistent with ancient selection events.

## Results

### Testing *Hmr* for Functional Divergence

**Figure 2:**
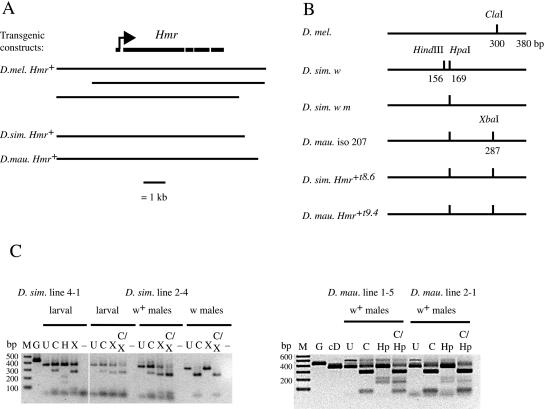
Structure and Expression of Sibling-Species *Hmr^+^* Transgenes (A) Diagram of *Hmr^+^* transgenic constructs. The *Hmr* gene structure is shown, with the rightward arrow indicating the predicted translation start site. Sibling-species constructs used in this study are shown, together with D. melanogaster constructs previously shown (Barbash et al. 2003) to be *Hmr^+^.* (B) Restriction map of RT-PCR fragments spanning part of exons 3 and 4, showing diagnostic restriction site polymorphisms found in the transgenic alleles and the stocks used to assay them. The D. melanogaster map corresponds to both the *Hmr^1^* and *In(1)AB* rescue alleles, as well as all D. melanogaster alleles from our population sample. (C) RT-PCR products from interspecific hybrids. Hybrids were from the crosses described in Table 1. RNA was collected from 48- to 72-h-old larvae and 2- to 4-d-old adult males. Note that larval samples contain RNA from males and females, half of whom carry the sibling-species *Hmr^+^* transgene. The portion of the PCR product derived from the transgenes is that digested by XbaI or HpaI for the D. simulans and D. mauritiana transgenes, respectively. M, 100-bp ladder marker; G, undigested PCR from an *Hmr* genomic clone (this product contains a 59-bp intron); cD, undigested PCR from an *Hmr* cDNA clone. The following are all RT-PCR products: U, undigested; C, ClaI-digested; H, HindIII-digested; X, XbaI-digested; C/X, ClaI- and XbaI-digested; Hp, HpaI-digested; C/Hp, ClaI- and HpaI-digested; –, control containing no reverse transcriptase. Note that undigested lanes (U) contain half the amount of DNA as digested samples.

In the absence of available *Hmr* mutations in the sibling species, we chose an alternative approach to test whether *Hmr* has functionally diverged by introducing cloned copies of D. simulans and *D. mauritiana Hmr^+^* into D. melanogaster (Figure 2). It has been shown previously that increasing *Hmr^+^* activity using *P*-element transgenes containing *D. melanogaster Hmr^+^* suppresses the hybrid-rescuing activity of *Hmr^1^* and *In(1)AB* mutations (Barbash et al. 2003). Those experiments can be interpreted as showing that the wild-type *D. melanogaster Hmr^+^* kills hybrids, with the *Hmr^1^* and *In(1)AB* mutations rescuing hybrids by reducing or eliminating *Hmr^+^* activity. The transgenic copies of *D. melanogaster Hmr^+^* thus suppress rescue by increasing *Hmr^+^* activity in hybrids. We therefore asked whether or not sibling-species *Hmr^+^* has the same activity. We reasoned that if *Hmr* is functionally diverged between the species, as in Figure 1B, then transgenes containing sibling-species *Hmr^+^* would not have the property of suppressing hybrid rescue. On the other hand, if *Hmr* is not functionally diverged, as in Figure 1C, then these sibling-species constructs would have phenotypic properties similar to the *D. melanogaster Hmr^+^* transgenes and suppress hybrid rescue.

**Table 1:**
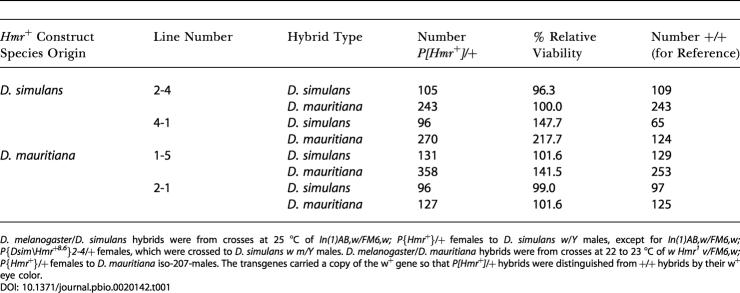
Viability of Hybrid Males Carrying D. simulans or *D. mauritiana Hmr^+^* Transgenes

D. melanogaster/D. simulans hybrids were from crosses at 25 °C of *In(1)AB,w/FM6,w; P{Hmr* ^+^}/+ females to D. simulans *w*/*Y* males, except for *In(1)AB,w/FM6,w; P{Dsim\Hmr^+8.6^}2-4*/+ females, which were crossed to D. simulans *w m/Y* males. D. melanogaster/D. mauritiana hybrids were from crosses at 22 to 23 °C of *w Hmr^1^ v/FM6,w; P{Hmr^+^}*/+ females to D. mauritiana iso-207-males. The transgenes carried a copy of the w^+^ gene so that *P[Hmr^+^]*/+ hybrids were distinguished from +/+ hybrids by their w^+^ eye color

To maximize the chances that the sibling-species transgenic lines would function normally, we made constructs that are similar to the largest *D. melanogaster Hmr^+^* transgenic construct and that exceed the minimal *Hmr^+^* region previously defined (see Figure 2A). We assayed two independent transformants each of *D. simulans Hmr^+^* and *D. mauritiana Hmr^+^* for suppression of hybrid male rescue by *Hmr^1^* in D. melanogaster/D. mauritiana hybrids and by *In(1)AB* in D. melanogaster/D. simulans hybrids (Table 1). In all cases hybrid males heterozygous for a sibling-species transgene were at least as viable as their brothers without the transgene. Some crosses showed an excess of transgene-carrying hybrids, which might suggest that the transgenes actually increase the effectiveness of hybrid rescue, perhaps by interfering with the pathway of *Hmr^+^*-dependent lethality. This possibility requires further investigation, but we note that such a hypothetical effect must be minor because the sibling-species *Hmr^+^* transgenes by themselves do not rescue hybrid males. We also assayed the *D. simulans Hmr^+^* transformants for suppression of *In(1)AB*-dependent rescue of hybrid female sterility (Barbash and Ashburner 2003). In contrast to the complete suppression associated with *D. melanogaster Hmr^+^* transgenes (Barbash et al. 2003), we found that our *D. simulans Hmr^+^* transgenes had little or no effect on egg counts in D. melanogaster/D. simulans hybrids. *In(1)AB,w/X_sim_,w m* females heterozygous for the insertion *P{Dsim\Hmr^+t8.6^}2-4* averaged 10.0 ± 8.9 eggs (*n* = 41) while their non-transgene-carrying sisters averaged 8.0 ± 12.4 eggs (*n* = 28). Using a second, independent transgenic line, we found that *In(1)AB,w/X_sim_,w* females heterozygous for the insertion *P{Dsim\Hmr^+t8.6^}4-1* had 12.2 ± 13.7 eggs (*n* = 24) while their non-transgene-carrying sisters had 17.7 ± 17.3 eggs (*n* = 31).

RT-PCR analysis demonstrated that the *Hmr^+^* transgenes are expressed (Figure 2C). These results show that the sibling-species alleles of *Hmr^+^* have no phenotypic effect in species hybrids and strongly support the conclusion that *Hmr* has functionally diverged between the D. melanogaster and sibling-species lineages, demonstrating that *Hmr* meets the criteria for being a D-M HI gene as diagrammed in Figure 1B.

### *Hmr* Divergence among *Drosophila* Lineages

It has been shown previously that *Hmr* has a high level of average divergence per nonsynonymous (amino-acid replacement) site *(D_N_)* between D. melanogaster and its three sibling species (Barbash et al. 2003). To understand this divergence in the context of genome-wide evolution, we calculated divergence of *Hmr* between D. melanogaster and D. simulans and compared it to compiled datasets containing over 250 genes from these two species (Begun 2002; Betancourt and Presgraves 2002). Pairwise comparisons revealed that *Hmr* has one of the highest levels of nonsynonymous divergence (0.089); only four other non–*Accessory gland protein (Acp)* genes have a higher nonsynonymous divergence, and two of these include expressed sequence tag comparisons less than 350 bases in length. The remaining two loci, both X-linked, are *mei-218* and *Odysseus*. In contrast, the average divergence per synonymous site *(Ds)* of 0.110 for *Hmr* is lower than the mean value of 0.125 for this dataset (again excluding *Accessory gland protein* genes).

The exceptional divergence of *Hmr* raises a number of important questions: (1) Is divergence high on the lineage of either D. melanogaster or its sibling species, or both? (2) Was the divergence on either or both lineages caused by positive selection consistent with the time scale of speciation? (3) Can we identify specific regions and codons subject to positive selection on these lineages, and how does this compare to the few other known candidate speciation genes? (4) Is divergence on one or both lineages potentially responsible for causing the HI phenotype of *Hmr*?

**Figure 3:**
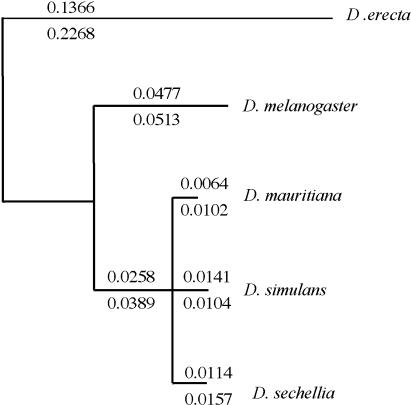
Maximum-Likelihood Estimates of *Hmr* Divergence among *Drosophila* Lineages Estimates of the number of changes per nonsynonymous site *(D_N_)* are shown above each lineage, and the number of changes per synonymous site *(D_S_)* are shown below each lineage, calculated separately for each branch. The coding region of *Hmr* is 1,390 to 1,427 amino acids long in the five species. *D_N_*/*D_S_* ratios differ significantly among branches as tested by the methods of Nielsen and Yang (1998). A model where all *D_N_*/*D_S_* ratios were free to vary along all branches (Model 2 [M2]) fit the data better than a model with a fixed *D_N_*/*D_S_* ratio for all branches (M1) (2Δ| = 308, *p* < 0.0001, chi-square distribution), as did a model where *D_N_/D_S_* ratios for the D. melanogaster lineage and the lineage leading to the sibling species differed from the rest of the tree (local clock, 2Δ| = 292, *p* < 0.0001). This suggests that most of the heterogeneity in the *D_N_*/*D_S_* ratio among branches of the phylogeny is due to an elevated ratio for the lineages leading from the ancestor of D. melanogaster and the sibling species. The tree is unrooted and we assume a trifurcation among the sibling species.

We first addressed whether *Hmr* began to diverge rapidly after the D. melanogaster–sibling-species divergence by isolating and assembling an outgroup *Hmr* orthologous sequence from the D. melanogaster subgroup species D. erecta, which is estimated to have diverged from D. melanogaster between 6 and 15 million years ago (Powell 1997). The maximum-likelihood estimate of *D_N_* between D. melanogaster and *D. erecta Hmr* was 0.166, higher than the mean value of 0.057 in a survey of 53 D. erecta genes (Bergman et al. 2002). However, the *D_N_*/*D_S_* ratio was 0.556, consistent with a more selective constraint on nonsynonymous sites than on synonymous sites. In contrast, we found that the D. melanogaster lineage and the lineage leading to the sibling species both exhibited elevated levels of nonsynonymous divergence relative to synonymous divergence (Figure 3). This observation suggests that the rate of amino-acid evolution at *Hmr* accelerated after the divergence of D. melanogaster from its sibling species. We also detected accelerated divergence along the sibling-species lineages, but because of the trichotomy for *D. simulans, D. sechellia,* and D. mauritiana, these branch lengths have little confidence. Furthermore, this divergence appears to be irrelevant with respect to the HI phenotype because we showed above that transgenic copies of *Hmr^+^* from both D. simulans and D. mauritiana have no effect on hybrid viability (Table 1).

### Positive Selection at *Hmr*

We next asked whether *Hmr* nonsynonymous substitution rates are elevated along the D. melanogaster and sibling-species lineages because of relaxed selective constraints (e.g., McAllister and McVean 2000) or positive selection. Polymorphism data from populations in concert with interspecific divergence data allow one to detect departures from neutrality and to estimate the time in the past when selection events occurred. If the divergence of *Hmr* was due solely to a relaxed selective constraint (neutral processes), then one would expect that the ratio of nonsynonymous to synonymous divergence between species would be similar to the ratio of nonsynonymous to synonymous polymorphisms found within these species (McDonald and Kreitman 1991).

To test this we collected polymorphism data from 14 D. melanogaster and seven *D. simulans Hmr* alleles. The average nonsynonymous *(π_N_)* and synonymous *(π_S_)* polymorphism per site within species was moderately low in both D. melanogaster (*π_N_* = 0.0017; *π_S_* = 0.0052) and D. simulans (*π_N_* = 0.0060; *π_S_* = 0.0123) relative to other loci (Andolfatto 2001). However, in contrast to the neutral expectation, the ratio of nonsynonymous to synonymous substitutions was found to be in significant excess of the ratio of nonsynonymous to synonymous polymorphisms (Table 2). When mutations were polarized along both lineages with respect to *D. erecta,* excess nonsynonymous changes were observed along both the D. melanogaster lineage and the D. simulans lineage. These results are highly indicative of positive selection acting in both the D. melanogaster and D. simulans lineages at coding positions in *Hmr*. The same tests performed between D. melanogaster or D. simulans and D. erecta were not significant, reinforcing our inference that selection occurred along the D. melanogaster and sibling-species lineages and not along the D. erecta lineage.

**Table 2:**
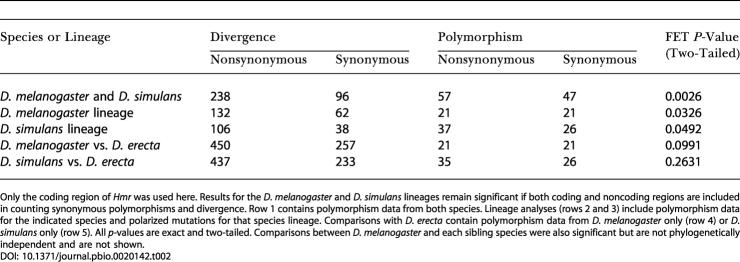
MK Tests for Deviations from Neutrality at *Hmr*

Only the coding region of *Hmr* was used here. Results for the D. melanogaster and D. simulans lineages remain significant if both coding and noncoding regions are included in counting synonymous polymorphisms and divergence. Row 1 contains polymorphism data from both species. Lineage analyses (rows 2 and 3) include polymorphism data for the indicated species and polarized mutations for that species lineage. Comparisons with D. erecta contain polymorphism data from D. melanogaster only (row 4) or D. simulans only (row 5). All *p-*values are exact and two-tailed. Comparisons between D. melanogaster and each sibling species were also significant but are not phylogenetically independent and are not shown

### Significant Regional Variation in Polymorphism and Divergence across *Hmr*

**Figure 4:**
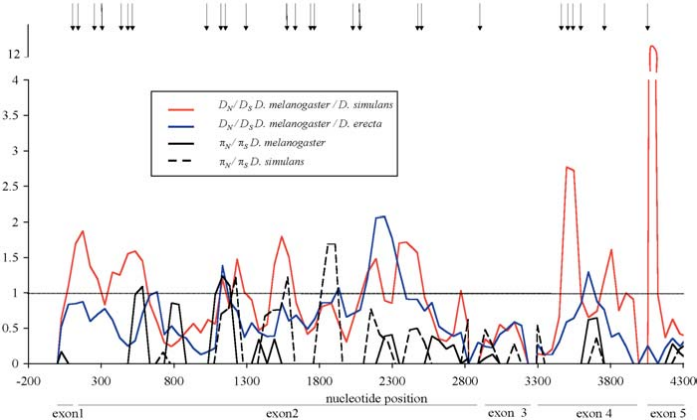
Sliding Window Analysis of *Hmr* Divergence and Polymorphism Calculations were made with a window size of 150 nucleotides and a step size of 50 nucleotides. Nucleotide position 1 on the *x*-axis is the start of the coding sequence, and the last position is the stop codon. The dashed line indicates where the ratio is one. Arrows at the top indicate the positions of codons identified as being under positive selection in Figure 5. Exon boundaries are indicated below the *x*-axis with horizontal bars. A repeatability analysis (Smith and Hurst 1998) revealed that polymorphism ratios for each window were not correlated (*p* = 0.43) with divergence ratios between D. melanogaster and D. simulans.

Polymorphism and divergence vary substantially for the separate exons and the DNA binding domains. In contrast to the neutral expectation, we found that there is highly significant heterogeneity in the ratio of polymorphism to divergence across the five exons of *Hmr* (Hudson-Kreitman-Aguade test [HKA test; Hudson et al. 1987]; *p* = 0.025; 10,000 simulations performed). This significant variation across the locus can be further refined by examining the ratio of nonsynonymous to synonymous variation across the locus and is visually displayed for D. melanogaster and D. simulans using sliding window analyses in Figure 4. These plots reveal multiple regions that have very high *D_N_*/*D_S_* ratios and low *π_N_*/*π_S_* ratios, in other words, much more amino-acid divergence than polymorphism. These contrasting ratios do not suggest that regional variation in substitution rates among the two classes of sites is due solely to mutation (and drift) but rather suggest that selection is contributing to the divergence pattern across the gene. Although this sliding window plot is highly suggestive of selection, we wished to obtain independent and statistically supported evidence for regional selection. We therefore tested rates of divergence relative to polymorphism for each exon in a manner similar to the McDonald-Kreitman test (MK test) described in the previous section. The data were partitioned a priori using each exon as a unit (rather than picking an arbitrary window size). Given the size of the sampled region, there is considerable power to address regional variation. These MK tests for each exon revealed that the fourth and fifth exons of *Hmr* appear to contribute the most to the overall MK test (Fisher's Exact Test [FET] and Bonferroni correction; exon 4, *p* = 0.0004; exon 5, *p* = 0.013). It is interesting to note that these regions are not homologous to any other proteins known in D. melanogaster (or in any other species), including other MADF domain–containing proteins. We conclude that multiple regions of *Hmr* show strong evidence for positive selection.

We also observed that the flanking regions of the *Hmr* locus appear to have evolved rapidly since D. melanogaster and the sibling species diverged, relative to both synonymous coding sites and introns. The ratios of divergence to polymorphism between D. melanogaster and D. simulans for the 5′ and 3′ flanking regions combined (including the UTRs) are significantly different from those of the coding and intron regions (3 × 2 FET; *p* < 0.0001) (Table 3), but this significant difference is not observed between D. simulans and D. mauritiana or D. sechellia (FET; *p* = 0.543 and *p =* 0.783, respectively). These observations suggest that adaptive fixations have occurred at a number of noncoding sites.

**Table 3:**
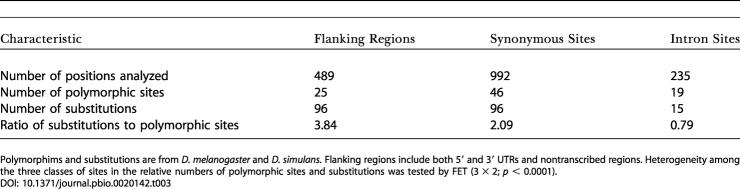
Observed Number of Replacement Substitutions Relative to Polymorphisms in *Hmr* for the Three Categories of Silent Sites

Polymorphims and substitutions are from D. melanogaster and *D. simulans.* Flanking regions include both 5′ and 3′ UTRs and nontranscribed regions. Heterogeneity among the three classes of sites in the relative numbers of polymorphic sites and substitutions was tested by FET (3 × 2; *p* < 0.0001)

**Figure 5:**
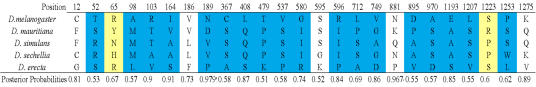
Phylogenetic Analysis of Positive Selection at Individual Codons Site-specific codon Model 8 (M8) in PAML was used to identify codons under selection. This model, which considers a discrete distribution of *D_N_*/*Ds* values plus a “selection category”—*D_N_*/*D_S_* values greater than one—fit the data better than a neutral model (M1) (2Δ| = 25.43, *p* < 0.001). Codons listed are those with *p*-values from posterior distributions greater than 0.5. The positions of these codons are also shown in Figure 4. Because D. melanogaster is incompatible with all three of its sibling species, we expect that *Hmr* codons involved in HI must be different between *D. melanogaster Hmr* and all three sibling-species alleles. Codons that fit a model of incompatibility between *Hmr_mel_* and *A_sib_* are shaded blue, and those that fit a model of incompatibility between *Hmr_anc_* and *A_sib_* are shaded yellow (see Discussion). The five remaining codons (unshaded) are identical between D. melanogaster and at least one of the sibling alleles and are thus excluded from both models.

We next asked whether we could refine the targets of selection to the level of individual codons and correlate such data with different models of HI evolution (Figure 5). Phylogenetic approaches similar to those used in Figure 3 have been designed to detect recurrent positive selection at individual codons among species (Yang and Nielsen 2002). We reasoned that the large amount of divergence at *Hmr* might offer a unique opportunity to apply these approaches to identify codons that exhibit positive selection and that could be contributing to HI. We identified 25 amino-acid positions that may have diverged due to selection, 20 of which fit possible models for the evolution of the D. melanogaster–sibling-species incompatibility (Figure 5). Many of these codons map to regions with peak *D_N_*/*D_S_* values (see Figure 4). We conclude that multiple regions show evidence of positive selection and may have contributed to the functional divergence of *Hmr* between D. melanogaster and its sibling species.

### Selection Events Are Ancient

Loci directly involved in reproductive isolation may reflect the true species history of divergence more accurately than a “random” locus sampled from the genome, because these loci cease exchanging alleles among species earlier than other loci (Wang et al. 1997; Ting et al. 2000). Both reduced gene flow and adaptive fixations can remove shared ancient polymorphisms. D. melanogaster and D. simulans separated approximately 2 to 3 million years ago (Powell 1997), and many shared polymorphisms have been lost due to drift (Clark 1997). However, shared polymorphisms are still observed in these genomes: among 15 loci (Andolfatto and Przeworski 2000), we found that 5.2% and 3.5% of segregating sites in D. melanogaster and D. simulans, respectively, are segregating at the same positions (among all classes of sites). Based on these values we expected to find approximately three to four shared polymorphic sites for *Hmr* among the 54 segregating sites in D. melanogaster and 94 segregating sites in D. simulans, but instead found zero. Although the expectation of three to four shared polymorphisms is not a strong test, these findings are consistent with *Hmr* alleles not having been exchanged between these taxa since at or near the time of species divergence and/or with selection events having occurred throughout the *Hmr* gene and having removed recurrent mutations.

**Figure 6:**
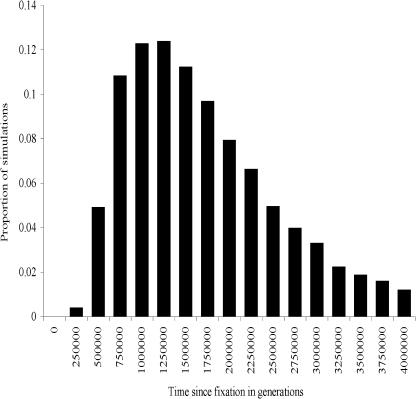
Posterior Distribution of Time (in Generations) Since the Most Recent Selective Sweep at *Hmr* for D. melanogaster Population size *(N)* is assumed to be 1 × 10^6^ for *D. melanogaster.* Samples were generated from the joint posterior distribution of five parameters of a selective sweep model assuming a selection event occurred sometime in the past at the *Hmr* locus, and from three summaries of polymorphism, including the number of segregating sites (54), Tajima's D (−0.61), the population recombination rate (4*Nr* = 43, where *N* = population size and *r* = per gene recombination rate; McVean et al. 2002) and the number of haplotypes (11). The data is least consistent with a selective sweep in the recent past and is most consistent with selective sweeps occurring more than *N* generations ago. If there are ten generations per year, this suggests that the last selective sweep occurred at least 100,000 years ago. Data for D. simulans are not shown, as the population structure for the D. simulans Wolfskill populations we sampled would inflate estimates through increasing marginal frequencies of segregating sites (Wall et al. 2002).

We have shown by several methods that the elevated rate of amino-acid divergence at *Hmr* is due to positive selection. These findings raise the critical question of whether selection events occurred recently or at the time of speciation. The number of years separating D. melanogaster and the sibling-species lineage makes this a challenging task. We therefore concentrated on the more tractable problem of looking for evidence to exclude the possibility that *Hmr* divergence reflects the action of contemporary or recent selection. In other words, while it may not be possible to prove that selection occurred at *Hmr* at the time of speciation, it is nevertheless important to demonstrate that available data are, at the least, not inconsistent with more ancient selective events. Examination of the frequency spectrum of polymorphism within both D. melanogaster and D. simulans showed no evidence of recent sweeps, as the distribution of per-site heterozygosities did not differ from expectations of neutrality (Tajima's D is −0.61 and 0.75, respectively, which is not significant). However, the power for detecting a selective sweep with this method is not high (75%) even if the event occurred in the very recent past, and diminishes to 25% as early as 0.8*N* generations ago (where *N*= population size) (Przeworski 2002). Because we have prior knowledge from the analyses above that selection events occurred at *Hmr,* we reasoned that we could apply more powerful Bayesian approaches that use not only frequency spectrum information but other summary statistics of polymorphism and recombination to estimate the time at which these events happened (Przeworski 2003). To estimate simultaneously the time and strength of selection events we conditioned on three summary statistics of the data: the polymorphism frequency spectrum (Tajima's D), the number of segregating sites, and the number of haplotypes. Prior distributions were chosen based on estimates of recombination, mutation rates, and effective population size. The most recent common ancestor for an *X*-linked locus is on average 3*N* generations ago. We determined that in *D. melanogaster,* a recent selective sweep (within the last 0.25*N* generations) is clearly not consistent with the polymorphism data, whereas adaptive mutations occurring more than *N* generations ago are consistent with the polymorphism data, and selective sweeps more than 1.5*N* generations ago are possible (Figure 6). Similarly, the marginal distribution of selection coefficients suggests that the strength of selection is inconsistent with small selection coefficients and recent selective sweeps (data not shown). Together, these results show that given our knowledge that selection has occurred at a large number of codons at *Hmr,* the last selection event occurred in the distant past. These procedures have been applied to infer selection events associated with human evolution (Przeworski 2003) but have not previously been applied to HI loci. Estimating the time of fixation events is integral to determining whether selection events that are found at HI loci are consistent with the known time frame of speciation or might merely reflect ongoing contemporary selection.

## Discussion

We have shown here that transgenes carrying D. simulans or *D. mauritiana Hmr^+^* have no effect on hybrid fitness, in contrast to the strong deleterious effects previously observed with *D. melanogaster Hmr^+^.* These transgenic experiments demonstrate that *Hmr* has functionally diverged between D. melanogaster and its sibling species and thus meets the experimental criteria defined by the D-M model. To our knowledge this is the first demonstration of functional divergence for a D. melanogaster HI gene. Although transgenic technologies have limitations—for example, genes with large and complex regulatory regions may not function correctly when transformed—they have the clear advantage of providing unambiguous analyses of single genes. Transgenics was previously used (Winkler et al. 1994) to show that the *Xmrk-2* gene from the platyfish Xiphophorus maculatus causes HI (although for technical reasons it was assayed in the related fish Oryzias latipes rather than the actual hybridizing species X. helleri [Weis and Schartl 1998]). Functional divergence is inferred in this case because *Xmrk-2* (also called ONC-X*mrk*) is a gene duplication that is present in X. maculatus but appears to be absent from X. helleri). Functional divergence can also be inferred in other HI systems where multigene regions are transferred between species by repeated backcrossing. This approach has been used to show that *Odysseus (Ods)* has functionally diverged: X-chromosome introgressions from D. mauritiana into D. simulans that contain the *D. mauritiana Ods* region are sterile, while related introgressions that lack *D. mauritiana Ods* remain fertile (Ting et al. 1998).

Both *Hmr* and *Ods,* as well as *Nup96,* which has recently been implicated in causing lethality in D. melanogaster/D. simulans male hybrids (Presgraves et al. 2003), show high rates of divergence for part *(Ods* and *Nup96)* or most *(Hmr)* of their coding regions. If generally true, this finding may reflect the fact that a high rate of substitution is required in order to generate a rare HI-causing mutation. Alternatively, it may be an indication that the combined effects of multiple substitutions are required to generate an HI allele. Our analysis of *Hmr* suggests two additional characteristics of HI genes. One is that while the D-M model requires divergence along only one lineage in order to generate HI, we have strong statistical support for accelerated divergence of *Hmr* along both lineages since D. melanogaster and the sibling species split.

One consequence of this extensive divergence is that it becomes unexpectedly complicated to identify the divergent codons in *Hmr* that are candidates for causing the incompatibility. The simple model shown in Figure 1B, where HI is caused by the interaction of *Hmr_mel_* and *A_sib_,* implies that any codons in *Hmr_mel_* that have diverged from the ancestral allele could potentially be causing the incompatibility; these codons could also diverge in the sibling-species alleles as long as they remain different from the D. melanogaster allele. There are 137 amino acids in *Hmr* (plus two sites of D. melanogaster–specific insertions) that fit these criteria. However, considering the extensive divergence we have detected at *Hmr* along both lineages, a more realistic model is shown in Figure 1D, where both *Hmr* and gene *A* go through multiple changes in both D. melanogaster and the sibling species. This model raises a possibility described by Muller (1942), namely, that a derived allele may become incompatible with an ancestral allele. For example, *A_sib_* may become incompatible with *Hmr_anc_,* which means that *Hmr_mel_* will also be incompatible if it retains the interacting residues present in *Hmr_anc_.* Since *Hmr_sib_* must be compatible with *A_sib_,* the candidate codons are those where *Hmr_sib_* differs from *Hmr_anc_* and *Hmr_mel_,* and *Hmr_mel_* remains identical to *Hmr_anc_.* There are 49 amino acids that fit this model, all of which are different from those identified in the previous model. Distinguishing between these models may be possible by using our transgene assays on *Hmr^+^* constructs containing site-directed changes at candidate amino acids.

Divergence due to positive selection is a second striking characteristic of *Hmr* as well as *Ods* and *Nup96.* Adaptive fixations and reproductive isolation at *Hmr* have clearly shaped the pattern of polymorphism relative to divergence and swept away any shared polymorphisms that may have been present. This characteristic raises the general question of what forms of selection are responsible and whether genes involved in certain traits or phenotypes that are under strong directional selection may preferentially contribute to HI. In allopatric models, where speciation occurs between two populations in geographical isolation, HI between species is strictly a secondary consequence of divergence of HI genes that has occurred within each species. The target of selection must therefore be sought by looking at gene function within species. We do not yet know the function of *Hmr; Hmr^1^* mutants are viable and fertile, but this allele is clearly hypomorphic (Barbash et al. 2003) and the null phenotype remains unknown. Because *Hmr* is expressed and causes HI in both sexes, however, it appears unlikely that it diverged under sexual selection. We do know that the pattern of variation in *Hmr* is not consistent with only weak selection events that have occurred recently. Rather, *Hmr* polymorphism is consistent with selective events occurring more than 0.5*N* generations ago and with a potentially large range of selection coefficients. Also unique to *Hmr* is that we have shown that it is likely to have been subject to multiple selective sweeps in both the D. melanogaster and sibling-species lineages and that the signal of positive selection comes from multiple sites and regions.

We have argued here that experimental demonstrations of functional divergence are required to prove that a gene is a bona fide HI locus. While man has undoubtedly been aware of the phenomenon of plant and animal HI for thousands of years, and hundreds of examples have been described in the scientific literature, identifying the genes involved has progressed rather slowly, with candidates generally being discovered by either genetic mapping or suppressor screens. As genomic sequences become available for species closely related to model organisms such as *D. melanogaster,* we suggest that the characteristics of *Hmr,* including high levels of divergence due to positive selection, may provide an alternative means of identification, with comparative genomics being used to identify candidate HI genes.

## Materials and Methods

### 

#### Nomenclature

The subscripts *mel, sib,* and *anc* are used to designate genes from *D. melanogaster,* its three sibling species, and their (hypothetical) ancestor, respectively.

#### RT-PCR

RT-PCR was performed from total RNA as described in Barbash et al. (2003). Thirty-five or 40 cycles of PCR were performed in a 50-μl volume with the oligos 5′-AAATCGAATCGCTTGTTTGG-3′ and 5′-CTCGAGCGGATGGTAGCGCAC-3′ at an annealing temperature of 61 °C. Two reactions per template were processed with QIAquick PCR Purification (Qiagen, Valencia, California, United States), eluted in 50 μl of 10mM Tris-HCl (pH 8.0), ethanol precipitated in the presence of 10 μg of glycogen, and resuspended in 10 μl of 10mM Tris-HCl (pH 8.0). Two microliters of DNA was digested with the appropriate restriction enzyme and run on a 2% agarose/TAE gel.

#### Clones and constructs

Sibling-species *Hmr* constructs were derived from D. simulans and D. mauritiana lambda genomic libraries (Ting et al. 1998). A D. simulans phage clone was isolated using the *D. melanogaster Hmr* cDNA clone RE54143 (Barbash et al. 2003) as a probe. An approximately 3.8-kb BamHI/NotI fragment of phage DNA was cloned into pBSII KS(+) (Stratagene, La Jolla, California, United States) to create p88. The adjacent approximately 5.8-kb BamHI phage DNA fragment was then cloned into the BamHI site of p88 to create p89; the correct orientation was determined by PCR across the BamHI junction. Using an XbaI site near the end of the p89 insert, p92 was made by cloning the approximately 8.6-kb NotI/XbaI fragment from p89 into pCasper4. End sequencing of this construct demonstrated that it extends from approximately 2.8 kb 5′ of *Hmr* to approximately 1.05 kb 3′ of *Hmr*; this construct also contains the complete predicted coding region of *CG2124.* The formal designation of this construct is *P{w^+mc^ Dsim\CG2124^+t8.6^ Dsim\Hmr^+t8.6^= Dsim\Hmr^+t8.6^}.*

A D. mauritiana phage clone was isolated using a D. mauritiana PCR product from exon 2 of *Hmr* as a probe. The entire phage insert was cloned into the NotI site of pBSII KS(+) to make the p94 plasmid. An approximately 9.4-kb NotI/XbaI fragment of the p94 insert was cloned into the corresponding sites of pCasper4. End sequencing of the insert demonstrated that this construct extends from approximately 2.8 kb 5′ of *Hmr* to approximately 1.8 kb 3′ of *Hmr.* This construct also contains the complete predicted coding regions of *Rab9D* and *CG2124.* The formal designation of this construct is *P{w^+mc^ Dmau\Rab9D^+t9.4^ Dmau\Hmr^+t9.4^ Dmau\CG2124^+t9.4^=Dmau\Hmr^+t9.4^}.*

*D. erecta Hmr* was isolated by screening a gridded fosmid library (BACPAC Resource Center, Oakland, California, United States) with *D. melanogaster Hmr* cDNA clone RE54143 as a probe. An approximately 8-kb XbaI fragment was subcloned from fosmid 11D-19 into pBSII KS(+) and sequenced using a GPS-1 Genome Priming System (New England Biolabs, Beverly, Massachusetts, United States) and custom sequencing primers.

We also identified an *Hmr* ortholog from the more distant *D. pseudoobscura,* but an unalignable repetitive region in the second exon made unambiguous calculations of amino-acid and silent divergence impossible.

As is found in D. melanogaster, the ortholog of *CG2124* is located 5′ of *Hmr* in opposite orientation in both D. erecta and *D. pseudoobscura,* demonstrating that we have correctly identified the ortholog of *Hmr* in both of these species.

#### Population samples

Ten *D. melanogaster Hmr* alleles were sequenced from a collection of iso-X-chromosome stocks derived from isofemale lines collected in Zimbabwe (Begun and Aquadro 1993). Five *D. simulans Hmr* alleles were sequenced from a collection of highly inbred isofemale lines collected in Wolfskill, California, United States (Begun and Whitley 2000). *Hmr* alleles were amplified by PCR in five overlapping segments of approximately 1 to 1.5 kb and sequenced directly using Big Dye chemistry (version 3; Applied Biosystems, Foster City, California, United States). We also included in our analyses four D. melanogaster alleles and one each from the three sibling species reported previously (Barbash et al. 2003), as well as the D. simulans p92 clone described above. Our population data set contains the complete coding region of *Hmr* as well as 447 bases upstream and 326 bases downstream of the coding region.

#### Sequence analysis

Sequences were aligned using ClustalX. Phylogenetic analyses were performed with PAML (Yang 1997). Population genetic analyses were performed using C++ libraries from K. Thornton. Estimates of the population recombination rate were calculated using Ldhat (McVean et al. 2002). Exact *p-*values for FETs were derived using the R statistical package. The HKA test was performed using the HKA program by Jody Hey. Estimates of the posterior distribution for time in generations since the most recent selective sweep were estimated using the method of Przeworski (2003).

## Supporting Information

### Accession Numbers

The GenBank accession numbers of the genes discussed in this paper are *Hmr* coding region from D. erecta (AY568390), *Hmr* coding region from D. mauritiana contained in plasmid p94 (AY573924), *Hmr* coding region from D. simulans contained in plasmid p92 (AY568391), *D. melanogaster Hmr* alleles sequenced from iso-X-chromosome stocks derived from isofemale lines collected in Zimbabwe (AY568380–AY568389), and *D. simulans Hmr* alleles sequenced from highly inbred isofemale lines collected in Wolfskill, California, United States (AY568392–AY568396).

## Abbreviations

D-M: Dobzhansky-Muller
D_N_: average per-site nonsynonymous nucleotide divergence
D_S_: average per-site synonymous nucleotide divergence
FET: Fisher's Exact Test
HI: hybrid incompatibility
Hmr: *Hybrid male rescue;* HKA test
MK test: McDonald-Kreitman test
π_N_: average per-site nonsynonymous nucleotide polymorphism
π_S_: average per-site synonymous nucleotide polymorphism
